# Statistical analysis of microarray gene expression data from a mouse model of toxoplasmosis

**DOI:** 10.1186/1471-2105-12-S7-A19

**Published:** 2011-08-05

**Authors:** Shrikant Pawar, Cheryl D Davis, Claire A Rinehart

**Affiliations:** 1 Department of Biology, Western Kentucky University, Bowling Green, KY 42101, USA; 2 Bioinformatics and Information Science Center, Western Kentucky University, Bowling Green, KY 42101, USA

## Background

Toxoplasmosis, caused by the protozoan parasite *Toxoplasma gondii* is a major cause of morbidity and mortality in patients with AIDS and an important cause of miscarriage, stillbirth and congenital disease in newborns. Previous studies have provided evidence that dietary supplementation with vitamin E and selenium is harmful during experimental toxoplasmosis in mice, whereas a diet deficient in vitamin E and selenium results in decreased numbers of tissue cysts in the brain and dramatically reduced brain pathology. The overall goal of the present study was to determine the impact of dietary supplementation with antioxidants on gene expression in the brains of non-infected mice and in mice infected with *T. gondii* using microarray analysis. RNA was isolated from the brains of C57BL/6 mice, and an Agilent Oligo Whole Mouse Genome Microarray (Agilent Technologies, Inc.) was performed. A total of 48 chips were normalized by Z ratios and the Data Driven Harr Fisch Normalization methods. Differentially expressed genes were identified by applying thresholds to identify significant values and the results were compared between the normalization methods. These differentially expressed genes and their respective fold change ratios were used in Ingenuity Pathway Analysis (IPA) software to analyze the pathways involved with these genes.

